# Characteristics and risk factors of postoperative pneumonia after emergency surgery in tertiary general hospitals (2015–2024): a 10-year comparative analysis with elective surgery

**DOI:** 10.3389/fpubh.2026.1770408

**Published:** 2026-02-20

**Authors:** Zhenzhen Wu, Zhigang Zheng, Zhenghao Yu, Xiaoli Wu, Yunxi Liu, Mingmei Du, Hongwu Yao, Yanling Bai

**Affiliations:** 1 Department of Infection Prevention and Control, Zhengzhou Central Hospital Affiliated to Zhengzhou University, Zhengzhou, China; 2 Department of Disease Control and Prevention, The First Medical Center of Chinese PLA General Hospital, Beijing, China; 3 Fuxing Road Outpatient Department, Jingnan Medical District of Chinese PLA General Hospital, Beijing, China; 4 Medical School of Chinese PLA, Beijing, China

**Keywords:** elective surgery, emergency surgery, nosocomial infections, postoperative pneumonia, risk factor

## Abstract

**Objective:**

To analyze the characteristics and risk factors of postoperative pneumonia (POP) in emergency surgery at tertiary general hospitals, aiming to provide scientific evidence for early screening of high-risk populations and optimization of POP prevention strategies.

**Methods:**

A retrospective analysis was performed on POP data from emergency and elective surgery patients between 2015 and 2024. Infection characteristics were compared between the two groups.

**Results:**

Among 399,347 surgical patients, 2,478 (0.62%) developed POP. The incidence of POP was significantly higher in emergency surgery (2.28%) than in elective surgery (0.53%, *p* < 0.001). Emergency surgery departments with the highest POP incidence were neurosurgery (18.30%), cardiovascular surgery (12.24%), and thoracic surgery (9.04%). Predominant pathogens were Gram-negative bacteria, followed by fungi and Gram-positive bacteria. The detection rate of multidrug-resistant organisms (MDROs) was significantly higher in emergency surgery than in elective surgery (42.19% vs. 32.90%, *p* < 0.001), with the most common MDROs being pandrug-resistant *Acinetobacter baumannii* (PDR-AB), carbapenem-resistant Enterobacteriaceae (CRE), and pandrug-resistant *Pseudomonas aeruginosa* (PDR-PA). Multivariate analysis showed that preoperative hospitalization duration of 6–48 h was a protective factor for POP in emergency surgery; male sex, age ≥60 years, admission to ICU, ASA score ≥3, general anesthesia, surgical duration ≥3 h, and intraoperative blood loss >1,000 mL were independent risk factors (*p* < 0.05).

**Conclusion:**

Emergency surgery carries a significantly higher risk of POP than elective surgery. Special attention should be paid to patients undergoing neurosurgery, cardiovascular surgery, and thoracic surgery. For patients with identified risk factors, enhanced perioperative management and individualized preventive strategies are essential to reduce POP incidence and improve emergency surgery outcomes.

## 1 Introduction

Postoperative pneumonia (POP) is a prevalent surgical complication and major type of hospital-acquired infection, accounting for 50% of all hospital-acquired pneumonia cases (1, 2). It not only significantly prolongs hospital stays and increases medical costs but also elevates patient mortality risk (3). Literature indicates that high POP rates (32–39%) have been reported in thoracic and abdominal surgeries, particularly high-risk procedures such as cardiac surgery, esophagectomy, and lung transplantation (4), and POP incidence is significantly higher in emergency surgery patients than in elective surgery patients (5), rendering emergency surgery a key risk factor for POP. This higher risk may be attributed to several factors: emergency patients often present with severe, rapidly progressive conditions (e.g., trauma, septic shock, acute organ failure); key preoperative infection control measures—such as management of underlying diseases, respiratory function optimization, and prophylactic antibiotic administration—are frequently inadequately implemented; and prolonged postoperative immobility and delayed pulmonary function recovery further increase complication risk.

To our knowledge, most current studies focus on POP risk factors and prevention strategies in elective surgery, while systematic research on emergency surgery—where infection rates are higher—remains insufficient. Therefore, this study retrospectively analyzed data from 2015 to 2024 to investigate POP characteristics in emergency surgery, including infection rates, high-incidence departments, pathogen profiles, and potential risk factors. These characteristics were further compared with those of elective surgery, aiming to provide scientific evidence for POP prevention and control in emergency surgery.

Also, in In April 2024, the Hospital Management Institute of China’s National Health Commission issued the “Initiative to Strengthen Perioperative Infection Prevention and Control to Ensure Surgical Quality and Safety” (hereafter “Infection-Surgery Initiative”), which identifies gradual reduction of POP incidence as a primary objective. We conducted this research in order to provide data support for the “Infection-Surgery Initiative.”

## 2 Methods

### 2.1 Materials

**Figure 1:**
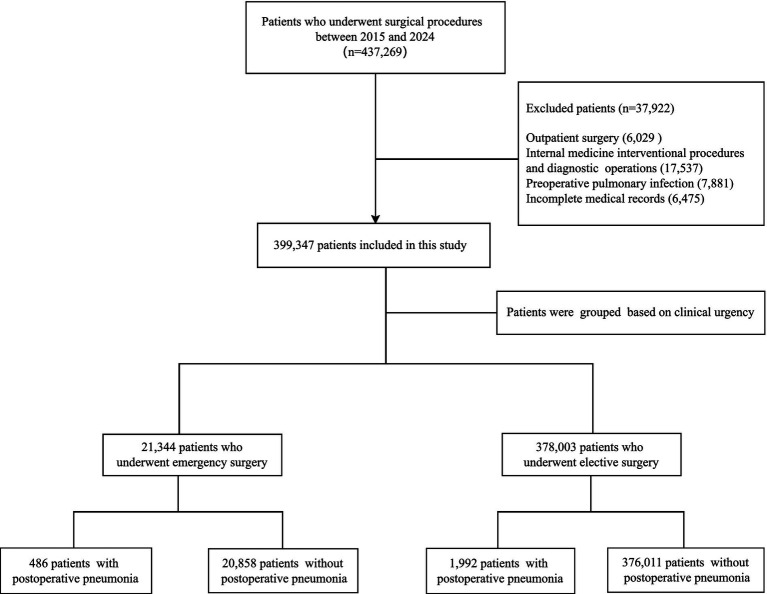
The flowchart of patient selection.

This study included all patients who underwent surgical procedures at a large tertiary hospital in Beijing, China, between January 1, 2015, and December 31, 2024. A total of 399,347 cases met the inclusion and exclusion criteria, among which 2,478 developed POP. The POP cohort consisted of 1,547 males and 931 females, with ages ranging from 1 to 94 years and a mean age of 60.07 ± 15.38 years. The flowchart of patient selection is shown in Figure 1. This study was approved by the hospital’s Ethics Committee (approval number: S2019-142-02).

Inclusion criteria: (1) Fulfilled surgical indications and underwent surgical procedures; (2) Hospitalization duration exceeded 48 h; (3) Complete medical records were available.

Exclusion criteria: (1) Outpatient surgery; (2) Internal medicine interventional procedures and diagnostic operations; (3) Preoperative pulmonary infection; (4) Incomplete medical records.

### 2.2 Data collection

Clinical data were obtained from the “Hospital Infection Real-time Monitoring System,” an established platform developed by the PLA General Hospital. This system integrates real-time data from the Hospital Information System (HIS), Laboratory Information System (LIS), Electronic Medical Record (EMR) and other systems. It generates automated alerts for potential infections, which were manually reviewed and validated by full-time infection control specialists to ensure diagnostic accuracy and exclude false positives. The following variables were extracted for analysis: baseline characteristics (age, gender, underlying diseases, and length of hospital stay), patients’ past medical history (primary pulmonary disease, hypertension, diabetes, and tumor), surgical data (surgical specialty, type of surgery, surgical duration, anesthesia method, intraoperative blood loss, and American Society of Anesthesiologists [ASA] score), and outcomes (infection onset time, clinical symptoms, and pathogens).

Patients were categorized into the emergency surgery group or the elective surgery group based on clinical urgency. For the purpose of this study, emergency surgery was defined as any surgical procedure performed on critically ill patients admitted to the emergency department that required immediate intervention, or on inpatients who required urgent surgery due to acute changes in their clinical condition. Conversely, elective surgery was defined as a pre-planned, scheduled procedure performed at a time convenient to both the patient and the surgeon.

### 2.3 Diagnostic criteria

The diagnosis of nosocomial infection is confirmed by infection control professional staff and clinicians based on the “CDC/NHSN Surveillance Definitions for Specific Types of Infections” (6) through bacteriological culture, routine examination, clinical symptom observation and imaging examination and other methods. POP is defined as pneumonia that develops within 30 days after surgery, including ventilator-associated pneumonia (VAP) (3).

Indicator calculation method: POP% = No. of new cases of pneumonia after surgery in emergency/elective inpatients/Number of surgeries in emergency/elective inpatients during the same period × 100%.

### 2.4 Pathogen identification and drug sensitivity testing

All specimens were routinely inoculated onto Mueller-Hinton agar and incubated at 35 °C for 24 h. Following isolation and purification, bacterial strains were identified, and antimicrobial susceptibility testing was performed using the VITEK-22 automated microbial analysis system (BioMérieux, Marcy-l’Étoile, France). Multidrug-resistant organisms (MDROs) were defined as bacteria resistant to three or more classes of antimicrobial agents. According to the provisional definition criteria for MDROs (7), the targeted strains primarily included methicillin-resistant *Staphylococcus aureus* (MRSA), methicillin-resistant *Staphylococcus epidermidis* (MRSE), pandrug-resistant *Acinetobacter baumannii* (PDR-AB), pandrug-resistant *Pseudomonas aeruginosa* (PDR-PA) and Carbapenem-resistant Enterobacteriaceae (CRE). For patients with multiple identical isolates detected from the same anatomical site, only the first isolate was included in the analysis.

### 2.5 Statistical analysis

Continuous data conforming to a normal distribution were expressed as mean and standard deviation; Continuous data with a non-normal distribution were presented as median and Interquartile Range (IQR), and comparisons between groups were performed using the Mann–Whitney U test. Categorical data were presented as frequencies and percentages, with comparisons between two groups performed using the *χ*^2^ test or Fisher’s exact test. All factors with statistical significance in univariate analysis were included in multivariate regression analysis using a binary logistic regression model. Odds ratios (OR) and 95% confidence intervals (CI) were calculated. A two-tailed *p* < 0.05 was considered statistically significant. All statistical analyses were conducted using SPSS software (version 27.0; SPSS Inc., Chicago, IL, USA).

## 3 Results

### 3.1 Incidence of POP in different years

A total of 399,347 surgical patients were included between 2015 and 2024, among whom 2,478 were diagnosed with POP, resulting in an overall POP incidence of 0.62%. The incidence of POP was significantly higher in emergency surgery (2.28%, 486/21,344) than in elective surgery (0.53%, 1,992/378,003; *χ*^2^ = 23.73, *p* < 0.001). These POP cases included 217 cases (44.65%) of VAP in the emergency group and 437 cases (21.94%) in the elective group (*χ*^2^ = 103.74, *p* < 0.001). Trend analysis revealed that the incidence of POP in elective surgery decreased from 0.76% in 2015 to 0.43% in 2024 (*χ*^2^ = 90.05, *p* < 0.001). In contrast, the incidence of POP in emergency surgery ranged from a low of 1.62% in 2015 to a high of 2.86% in 2019, with no statistically significant difference observed over the study period (*χ*^2^ = 14.79, *p* = 0.097), as shown in Table 1.

**Table 1:** Incidence of POP in emergency and elective surgery from 2015 to 2024.

Year	Emergency surgery			Elective surgery		
	No. of surgical cases	No. of infected cases	Infection rate (%)	No. of surgical cases	No. of infected cases	Infection rate (%)
2015	2,832	46	1.62	35,339	270	0.76
2016	2,708	61	2.25	36,043	252	0.70
2017	2,666	58	2.18	37,489	206	0.55
2018	2,406	59	2.45	42,235	168	0.40
2019	2,444	70	2.86	44,730	237	0.53
2020	1,416	40	2.82	25,648	114	0.44
2021	1,547	41	2.65	38,122	176	0.46
2022	1,715	30	1.75	30,793	179	0.58
2023	1,906	44	2.31	42,140	196	0.47
2024	1,704	37	2.17	45,464	194	0.43
Total	21,344	486	2.28	378,003	1,992	0.53
*χ* ^2^	90.05			14.79		
*P*	<0.001			0.097		

### 3.2 Distribution of POP occurrence time

**Figure 2:**
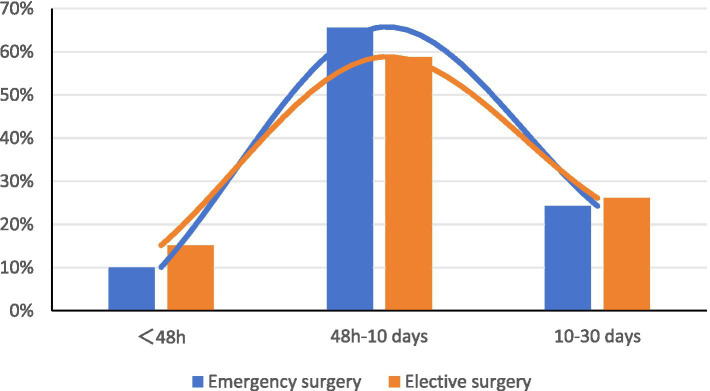
Distribution of POP occurrence time.

65.64% of POP cases in emergency surgery and 58.73% in elective surgery occurred 48 h–10 days after surgery. The median onset time was 6.21 days (3.65–9.71) for emergency surgery and 5.97 days (3.32–10.40) for elective surgery, with no significant difference (*Z* = −0.827, *p* = 0.408), as shown in Table 2 and Figure 2.

**Table 2:** Distribution of POP occurrence time in emergency and elective surgery.

Type of surgery	No. of cases of infection	Duration of postoperative infection				*Z*	*P*
		<48 h *n* (%)	48 h–10 days *n* (%)	10–30 days *n* (%)	Median (IQR)		
Emergency surgery	486	49 (10.08)	319 (65.64)	118 (24.28)	6.21 (3.65, 9.71)	−0.827	0.408
Elective surgery	1,992	302 (15.16)	1,170 (58.73)	520 (26.10)	5.97 (3.32, 10.40)		

### 3.3 Distribution of POP by department

POP was observed in 13 surgical departments. Except for Obstetrics and gynecology, the incidence of POP in emergency surgery was significantly higher than that in elective surgery across all departments (*p* < 0.001). Among emergency surgeries, the three departments with the highest POP incidence were Neurosurgery (18.30%), cardiovascular surgery (12.24%), and Thoracic surgery (9.04%). For elective surgeries, the top three departments were cardiovascular surgery (2.75%), neurosurgery (1.70%), and General surgery (0.75%). The largest disparity in POP incidence between emergency and elective surgery was noted in neurosurgery, with an approximately 11-fold difference, as shown in Table 3.

**Table 3:** The incidence of POP in different departments.

Department	Emergency surgery			Elective surgery			*χ* ^2^	*P*
	No. of surgical cases	No. of infections	Infection rate (%)	No. of surgical cases	No. of infection cases	Infection rate (%)		
Neurosurgery	1,191	218	18.30	30,038	512	1.70	1,382.67	<0.001
Cardiac Surgery	49	6	12.24	2,586	71	2.75	–	0.003^a^
Thoracic Surgery	188	17	9.04	30,297	153	0.51	–	<0.001^a^
Vascular surgery	328	29	8.84	9,643	33	0.34	–	<0.001^a^
General Surgery	2,184	102	4.67	63,005	474	0.75	367.0	<0.001
Breast Surgery	109	4	3.67	5,715	5	0.09	–	<0.001^a^
Orthopedic	911	24	2.63	63,459	243	0.38	–	<0.001^a^
Plastic Surgery	79	2	2.53	4,786	6	0.13	–	0.007^a^
Stomatology	188	4	2.13	18,068	38	0.21	–	<0.001^a^
Hepatobiliary surgery	2,181	46	2.11	49,987	302	0.60	71.43	<0.001
Otolaryngology	1,119	23	2.06	37,379	74	0.20	–	<0.001^a^
Urology	238	4	1.68	26,277	49	0.19	–	0.001^a^
Obstetrics and gynecology	12,441	7	0.06	32,842	32	0.10	1.78	0.182
Other	138	0	0.00	3,921	0	0.00	–	–

^a^Fisher’s exact probability method was used; − indicates that the data are not available.

### 3.4 Causative pathogens

A total of 2,766 pathogens were isolated from all POP patients, including 730 from emergency surgeries and 2,036 from elective surgeries. Gram-negative bacteria were the most commonly isolated pathogens in both groups (emergency vs. elective: 70.55% vs. 66.06%), with the primary causative pathogens being *Acinetobacter baumannii*, *Klebsiella pneumoniae*, and *Pseudomonas aeruginosa*, these results are summarized in Table 4.

**Table 4:** Distribution of pathogens detected in POP between emergency and elective surgeries.

Pathogen	Emergency surgery		Elective surgery	
	Strain	Composition ratio (%)	Strain	Composition ratio (%)
Gram-negative bacteria	515	70.55	1,345	66.06
*Acinetobacter baumannii*	185	35.92	432	32.12
*Klebsiella pneumoniae*	131	25.44	335	24.91
*Pseudomonas aeruginosa*	89	17.28	268	19.93
*Stenotrophomonas maltophilia*	46	8.93	124	9.22
*Enterobacter cloacae*	12	2.33	50	3.72
*Escherichia coli*	14	2.72	42	3.12
*Burkholderia cepacia*	17	3.30	32	2.38
*Mucilaginous Serratia*	8	1.55	27	2.01
Other negative bacteria	13	2.52	35	2.60
Gram-positive bacteria	81	11.10	318	15.62
Coagulase-negative staphylococci	54	66.67	207	65.09
*Staphylococcus aureus*	22	27.16	72	22.64
Other positive bacteria	5	6.17	39	12.26
Fungi	134	18.36	373	18.32
*Candida albicans*	44	32.84	159	42.63
*Candida tropicalis*	14	10.45	67	17.96
Candida smooth	28	20.90	57	15.28
Nearly smooth Candida	13	9.70	36	9.65
Filamentous fungi	17	12.69	26	6.97
Molds	11	8.21	18	4.83
Other fungi	7	5.22	10	2.68
Total	730	100	2,036	100

**Figure 3:**
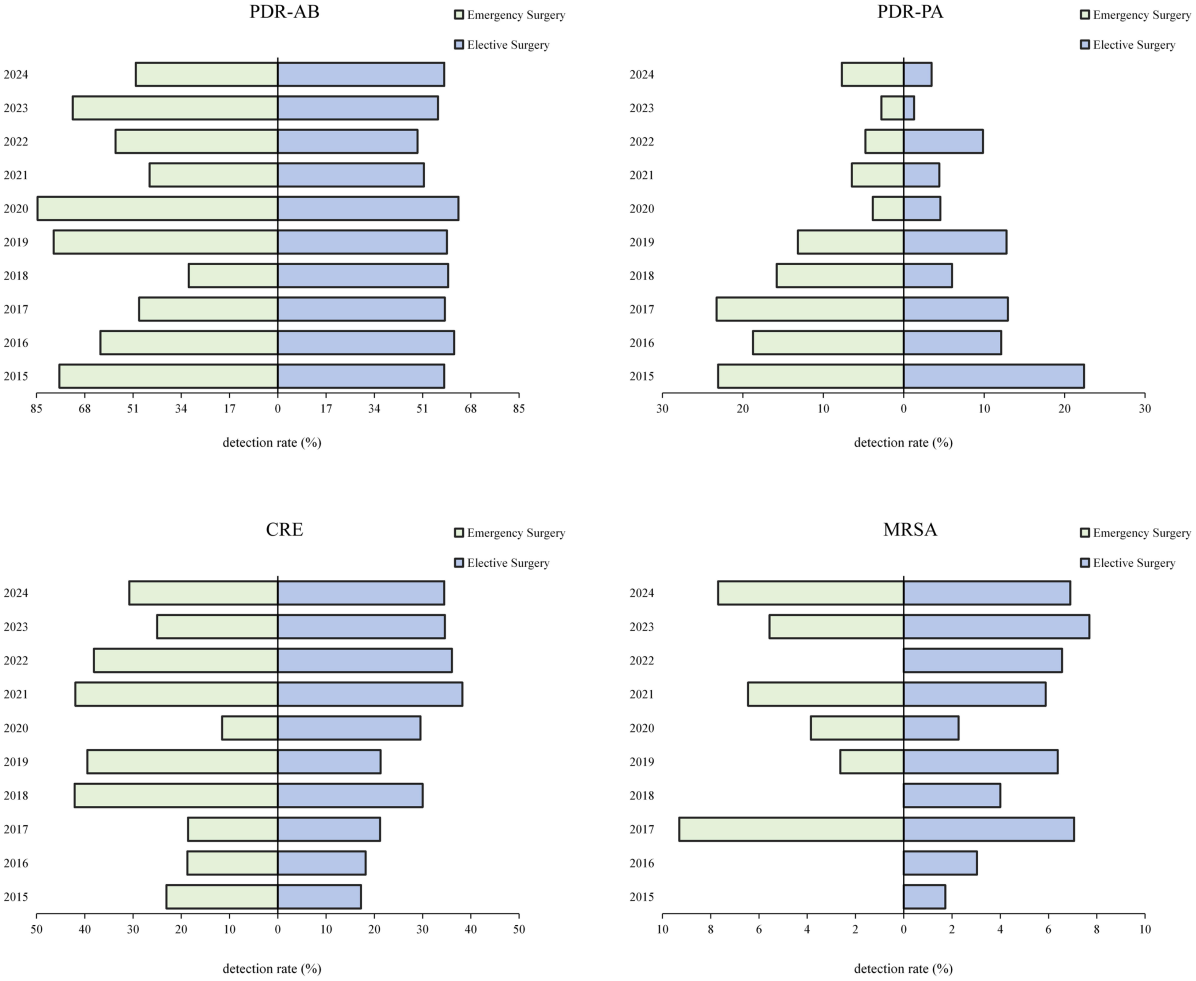
Distribution of MDROs detected in POP from 2015 to 2024.

In addition, MDROs were isolated from 308 specimens (42.19%) in emergency surgeries and 670 specimens (32.90%) in elective surgeries, with a statistically significant difference between groups (*χ*^2^ = 20.26, *p* < 0.001). The most prevalent MDROs were PDR-AB, CRE, and PDR-PA, as shown in Table 5. Ten-year trend analysis showed that PDR-AB had the highest detection rate in 2020 (emergency vs. elective: 84.62% vs. 63.64%), followed by a decline and then a re-increase in 2023. CRE exhibited the highest detection rate in 2021 (emergency vs. elective: 41.94% vs. 38.24%), after which it showed a downward trend, as shown in Table 5 and Figure 3.

**Table 5:** Distribution of MDROs detected in POP between emergency and elective surgeries.

MDROs	Emergency surgery		Elective surgery	
	Strain	Composition ratio (%)	Strain	Composition ratio (%)
PDR-AB	174	56.49	382	57.01
CRE	86	27.92	183	27.31
PDR-PA	34	11.04	61	9.10
MRSA	12	3.90	36	5.37
MRSE	2	0.65	6	0.90
VRE	0.00	0.00	2	0.30
Total	308	100	670	100

### 3.5 Risk factors of POP in emergency surgery

Univariate analysis revealed that sex, age, primary pulmonary disease, hypertension, diabetes, tumor, admission to ICU, preoperative hospitalization duration, ASA score, anesthesia method, surgical duration, and intraoperative blood loss were associated with POP occurrence in emergency surgery, with statistically significant differences (all *p* < 0.05), as shown in Table 6.

**Table 6:** Results of univariate analysis of risk factors for POP in emergency surgery.

Factors	POP group *n* (%)	Non-POP group *n* (%)	*χ* ^2^	*P*
Sex				
Female	181 (37.24)	11,064 (53.04)	47.572	<0.001
Male	305 (62.76)	9,794 (46.96)		
Age (years)				
<60	239 (49,18)	17,962 (86.12)	516.069	<0.001
≥60	247 (50.82)	2,896 (13.88)		
Primary pulmonary disease				
No	453 (93.21)	20,526 (98.41)	76.357	<0.001
Yes	33 (6.79)	332 (1.59)		
Hypertension				
No	394 (81.07)	19,482 (93.40)	112.790	<0.001
Yes	92 (18.93)	1,376 (6.60)		
Diabetes				
No	442 (90.95)	19,452 (93.26)	4.012	0.045
Yes	44 (9.05)	1,406 (6.74)		
Tumor				
No	399 (82.10)	19,477 (93.38)	94.356	<0.001
Yes	87 (17.90)	1,381 (6.62)		
Admission to ICU				
No	199 (40.95)	19,678 (94.34)	2,115.56	<0.001
Yes	287 (59.05)	1,180 (5.66)		
Preoperative hospitalization duration (h)				
≤6	238 (48.97)	7,929 (38.01)	127.794	<0.001
6–24	50 (10.29)	5,512 (26.43)		
24–48	24 (4.94)	2,801 (13.43)		
>48	174 (35.80)	4,616 (22.13)		
ASA score				
<3	101 (20.78)	18,241 (87.45)	1,746.651	<0.001
≥3	385 (79.22)	2,617 (12.55)		
Anesthesia method				
General anesthesia	475 (97.74)	14,047 (67.35)	201.711	<0.001
Non-general anesthesia	11 (2.26)	6,811 (32.65)		
Surgical duration (h)				
<3	273 (56.17)	19,468 (93.34)	944.289	<0.001
≥3	213 (43.83)	1,390 (6.66)		
Intraoperative blood loss				
<500	393 (80.86)	19,590 (93.92)	176.460	<0.001
500–1,000	60 (12.35)	1,025 (4.91)		
>1,000	33 (6.79)	243 (1.17)		

Multivariate logistic regression analysis demonstrated that preoperative hospitalization duration of 6–48 h was a protective factor for POP in emergency surgery. In contrast, male sex, age ≥60 years, admission to ICU, ASA score ≥3, general anesthesia, surgical duration ≥3 h, and intraoperative blood loss >1,000 mL were independent risk factors (p < 0.05), as shown in Table 7.

**Table 7:** Results of multifactorial logistic regression analysis of risk factors for POP in emergency surgery.

Factor	*B*	*P*	OR	95% CI	
Sex (male)	0.633	<0.001	1.883	1.534	2.311
Age (≥60 years)	0.698	<0.001	2.009	1.630	2.477
Admission to ICU	1.510	<0.001	4.528	3.612	5.678
ASA score (≥3)	1.792	<0.001	6.004	4.597	7.841
Anesthesia method (general anesthesia)	1.602	<0.001	4.962	2.654	9.276
Surgical duration (≥3 h)	0.707	<0.001	2.028	1.631	2.522
Intraoperative blood loss (>1,000 ml)	0.907	0.049	2.478	1.005	6.110
Preoperative hospitalization duration (6–24 h)	−0.702	<0.001	0.496	0.350	0.702
Preoperative hospitalization duration (24–48 h)	−0.487	0.039	0.039	0.614	0.387

## 4 Discussion

Studies have indicated that comprehensive interventions—including preoperative nutritional support and respiratory function training; intraoperative precise anesthesia, minimally invasive surgical techniques, and maintenance of physiological stability (e.g., body temperature, blood glucose level); and postoperative early extubation and mobilization—have effectively reduced POP incidence (8–10). In our study, the overall POP incidence was 0.63%, which is higher than the 0.22–0.43% reported by similar hospitals in China (11, 12), but lower than the 1.3–1.9% reported by Metersky et al. (13). Among POP cases, VAP accounted for 21.94% of cases in elective surgery and a much higher 44.65% in emergency surgery, emphasizing the need to strengthen management of postoperative patients on mechanical ventilation. Over the 10-year study period, the Elective surgery POP incidence decreased significantly (0.76% in 2015 vs. 0.43% in 2024). Notably, the incidence of POP in emergency surgery surged to 2.87% in 2020. This increase may be attributed to the impact of the Coronavirus Disease 2019 (COVID-19) pandemic, which caused a substantial reduction in surgical activity. As reported in a UK study (14), overall surgical activity decreased by 33.6% compared to pre-pandemic expectations. This reduction likely shifted the case load towards patients with more severe and critical conditions, thereby increasing the risk of POP. In addition, the incidence of POP in emergency surgery (2.28%) was significantly higher than that in elective surgery (0.53%), with no evidence of a downward trend over the decade. These differences may be associated with variations in pathogenesis, pathophysiological states, and perioperative management strategies. Emergency surgery patients often present with shock, trauma, or active infection, which can severely impair immune function. Moreover, limited preoperative preparation time restricts thorough infection screening, thereby increasing POP risk (15, 16). These results suggest that emergency surgery represents a key focus area requiring targeted interventions to reduce the incidence of postoperative pneumonia.

The incidence of POP varies significantly across different departments and surgical sites. Identifying the distribution of departments with high incidence of POP is crucial for formulating targeted prevention and control strategies. Among emergency surgeries, the top three departments with the highest incidence of POP were neurosurgery (18.30%), cardiovascular surgery (12.24%), and thoracic surgery (9.04%). For elective surgery, the top three were cardiovascular surgery (2.75%), neurosurgery (1.70%), and general surgery (0.75%). Neurosurgery showed the greatest disparity between emergency and elective surgeries, approximately 11-fold higher. This is associated with the severity of traumatic brain injury, spontaneous intracerebral hemorrhage, and acute epidural/subdural hematoma—the predominant pathologies in emergency neurosurgical patients—which often lead to impaired consciousness, thereby increasing the risk of aspiration. Moreover, it is often difficult to perform comprehensive preoperative respiratory assessments (e.g., aspiration risk evaluation) or preventive measures (e.g., oral hygiene) in emergency settings. Furthermore, these patients frequently require prolonged postoperative mechanical ventilation, all of which significantly increases the risk of pulmonary infection (17). Previous studies have indicated that POP is concentrated in thoracic and upper abdominal surgeries (18). Our dataset further demonstrated a high incidence of POP in cardiovascular surgical procedures. The major pathologies requiring cardiovascular surgery included acute Stanford type A aortic dissection, ruptured abdominal aortic aneurysm, and acute myocardial infarction—all of which necessitate urgent surgical intervention with extracorporeal circulation. Crucially, this circulatory support modality triggers pulmonary injury, where the severity of such damage increases proportionally with the duration of extracorporeal circulation (19). Patients undergoing thoracic surgery often transition from thoracic to abdominal breathing due to incision pain. This leads to persistent low tidal volume, reduced functional residual capacity, atelectasis, impaired coughing and expectoration ability, and retained airway secretions, increasing the risk of POP (20).

Pathogenic bacterial culture results exhibit high specificity and provide important guidance for the diagnosis and precise treatment of POP (21). However, the distribution of POP pathogens across different geographical regions and healthcare settings. A Japanese study (22) reported that the predominant pathogens isolated were *Pseudomonas aeruginosa*, *Staphylococcus aureus*, and *Enterococcus faecalis*, whereas research conducted by Xiang et al. (23) in China found that *Klebsiella pneumoniae*, *Escherichia coli*, and *Staphylococcus aureus* were the primary pathogens. This study revealed that Gram-negative bacteria accounting for 70.55% in emergency surgeries, *Acinetobacter baumannii*, *Klebsiella pneumoniae*, and *Pseudomonas aeruginosa* were the most commonly identified species, consistent with the findings of Xu et al. (24–26). The second was Fungal, *Candida albicans* were the predominant species. This is mainly attributed to the fact that Candida, as an opportunistic pathogen, can be aspirated into the lower respiratory tract and become a pathogenic agent causing POP when critically ill patients experience aspiration due to anesthesia, impaired consciousness, tracheal intubation, or indwelling nasogastric tubes.

The detection of MDROs in POP patients will further exacerbate the difficulty and complexity of clinical treatment. In this study, the detection rates of PDR-AB and CRE were the highest, consistent with results from previous studies (12, 27). Additionally, the detection rate of MDROs in emergency surgeries was significantly higher than in elective surgeries (42.19% vs. 32.90%). This disparity may be attributed to the critical condition of emergency surgery patients, immunosuppression under stress, incomplete infection screening in urgent preoperative situations, and the empirical use of broad-spectrum antibiotics, all of which increase the risk of colonization or infection by drug-resistant organisms. Furthermore, the distribution of MDROs detected over the 10-year study period revealed that PDR-AB had the highest detection rate in 2020 (84.62% in emergency surgeries vs. 63.64% in elective surgeries), decreased thereafter, and rose again in 2023, indicating that the situation of the prevention and control of PDR-AB is severe and repetitive, and that it is necessary to build a long-term management mechanism and dynamically adjust the prevention and control strategy. The highest CRE detection rates were observed in 2021 (41.94% in emergency surgeries vs. 38.24% in elective surgeries), followed by a subsequent decline. This is consistent with the current global trend of MDRO epidemiology (28). Therefore, when POP is suspected, pathogen samples should be collected and submitted in time and standardized in order to determine the appropriate use of antimicrobial agents.

The occurrence of POP is influenced by multiple factors, investigating the risk factors is essential for optimizing perioperative care, formulating individualized intervention strategies, and ultimately reducing the incidence of POP. Through large-scale data analysis, this study demonstrated that preoperative hospitalization duration of 6–48 h was a protective factor for POP in emergency surgery, whereas male sex, age ≥60 years, admission to ICU, ASA score ≥3, general anesthesia, surgical duration ≥3 h, and intraoperative blood loss >1,000 mL were independent risk factors(*p* < 0.05).

Studies have shown that performing surgery within 48 h, not only effectively reduces the risk of POP, but also lower the all-cause mortality risk by 19% in patients older than 60 years (29). The American Academy of Orthopaedic Surgeons (AAOS) recommends that hip fracture surgery should be performed as early as possible within 48 h of hospital admission (30). The risk of POP in male patients is 1.86 times higher than that in female patients, which may be related to physiological differences, immune status, and lifestyle. Some studies indicated that male airway is more susceptible to accumulate pathogens, whereas females tend to exhibit stronger innate immunity due to the influence of X chromosome genes and estrogen (31). In older adult patients, declining organ function and suffering from multiple comorbidities such as chronic cardiovascular and respiratory diseases, have reduced immunoglobulin levels and impaired cellular immune responses. These factors collectively impair resistance to pneumonia and associated with a higher incidence of POP, as shown in multiple studies (32, 33).

In addition, patients admitted to ICUs often have multiple underlying diseases and are in critical conditions. Invasive procedures such as mechanical ventilation and indwelling catheters are frequently performed during the therapeutic processes of postoperative. Moreover, the relatively enclosed and concentrated environment of the ICU increases the risk of POP. The ASA score, a widely accepted metric for preoperative patient assessment, reflects the overall health status and comorbidities of individuals. Studies have shown that ASA score is positively correlated with the risk of surgical complications, especially in patients with ASA score ≥3, the incidence of POP is significantly increased (34). General anesthesia can cause atelectasis and pulmonary collapse, with subsequent ventilation-perfusion mismatch contributing to hypoxemia. Moreover, the administration of opioid analgesics may suppress the respiratory center or induce respiratory muscle weakness. Collectively, these alterations impair respiratory function and effective cough ability, thereby increasing the risk of POP (35). Excessive intraoperative blood loss during surgery may result in significant depletion of immune factors and albumin. Severe anemia and hypoalbuminemia can lead to pulmonary edema, increased alveolar secretion, and reduced ventilatory efficiency (36). Epidemiological data (31) indicated that the incidence of POP in patients with ≥200 mL of intraoperative blood loss was 15.84%. However, this study found that blood loss exceeding 1,000 mL was a significant risk factor for POP, with greater blood loss correlating with increased severity. Our study demonstrated that surgical duration exceeding 3 h was associated with a higher incidence of pop. Prolonged surgery typically involves more extensive tissue manipulation and a heightened inflammatory response, which may trigger systemic inflammation (37) and thus necessitate the additional administration of antimicrobial agents.

## 5 Conclusion

The incidence of POP is significantly higher in emergency surgery patients than in elective surgery patients. Neurosurgery, cardiovascular surgery, and thoracic surgery are identified as high-risk departments that require increased vigilance. The pathogens of POP are primarily Gram-negative bacteria, and the detection rate of MDROs is higher in emergency surgery than in elective surgery, with particularly high detection rates for PDR-AB and CRE. Clinicians should use antimicrobial agents appropriately based on the characteristics of bacterial resistance. It is recommended to enhance perioperative management and implement individualized preventive strategies for high-risk populations in order to reduce the incidence of POP, and facilitate rapid recovery following emergency surgery.

## 6 Limitations

Despite the strengths of this study, including a sufficient sample size and comprehensive coverage of risk factors, several limitations should be acknowledged. As a single-center retrospective study, its findings are limited in generalizability due to institutional-specific patient characteristics and clinical practices. Additionally, Due to incomplete data documentation regarding smoking status and obesity, we were unable to investigate their potential impact on POP. Future multicenter prospective studies are needed to address these gaps.

## Data Availability

The raw data supporting the conclusions of this article will be made available by the authors, without undue reservation.
